# Differential binding of SARS-CoV-2 Spike protein variants to its cognate receptor hACE2 using molecular modeling based binding analysis

**DOI:** 10.6026/97320630017337

**Published:** 2021-02-28

**Authors:** Mirza Sarwar Baig, Enam Reyaz, Angamuthu Selvapandiyan, Anuja Krishnan

**Affiliations:** 1 Department of Molecular Medicine, School of Interdisciplinary Sciences & Technology, Jamia Hamdard, New Delhi - 110062, India

**Keywords:** SARS-CoV-2, Spike protein, hACE2, RBD, binding affinity, molecular docking, molecular modeling

## Abstract

The current emergence of novel coronavirus, SARS-CoV-2 and its ceaseless expansion worldwide has posed a global health emergency that has adversely affected the humans. With the entire world striving to understand the newly emerged virus, differences in morbidity and infection rate of SARS-CoV-2 have been observed across varied geographic areas, which have been ascribed to viral mutation and evolution over time. The homotrimeric Spike (S) glycoprotein on the viral envelope surface is responsible for binding, priming, and initiating infection in the host. Our phylogeny analysis of 1947 sequences of S proteins indicated there is a change in amino acid (aa) from aspartate (Group-A) to glycine (Group-B) at position 614, near the receptor- binding domain (RBD; aa positions 331-524). The two variants are reported to be in circulation, disproportionately across the world, with Group-A dominant in Asia and Group-B in North America. The trimeric, monomeric, and RBD of S protein of both the variant groups (A & B) were modeled using the Swiss-Model server and were docked with the human receptor angiotensin-converting enzyme 2 (hACE2) employing the PatchDock server and visualized in PyMol. Group-A S protein's RBD bound imperceptibly to the two binding clefts of the hACE2 protein, on the other hand, Group-B S protein's RBD perfectly interacted inside the binding clefts of hACE2, with higher number of hydrogen and hydrophobic interactions. This implies that the S protein's amino acid at position 614 near the core RBD influences its interaction with the cognate hACE2 receptor, which may induce its infectivity that should be explored further with molecular and biochemical studies.

## Background

In December 2019, the city of Wuhan, Hubei province of China, witnessed patients inflicted with severe atypical pneumonia and respiratory illness, reporting the first case of novel coronavirus (CoV-2) infection in December 2019 [1, 2]. Since then, it has grasped and restricted the entire globe, with nearly 85 million cases and more than 1.8 million fatalities as of the first week of January 2021 [3]. The genome of SARS- CoV-2 is around 30 kb, with a 5' end comprising orf1ab encoding orf1ab polyproteins, while the 3' end consists of genes encoding structural proteins, including spike (S), envelope (E), membrane (M), and nucleocapsid (N) proteins. The large replicase polyproteins pp1a and pp1ab are proteolytically cleaved into 16 putative nonstructural proteins (nsps). Structurally S proteins (1273 amino acids) are homotrimeric, with each monomer of about 180 kDa, consisting of two subunits, S1 and S2. SARS-CoV-2 has a functional polybasic (furin) cleavage site at the S1 - S2 boundary through the insertion of 12 nucleotides. The viral entry, a complex series of events, is prompted by the binding of the S1 subunit to the host cell receptor, that in turn, triggers priming cleavage by cellular proteases at S1/S2 domain, and activation cleavage at S2', followed by insertion of the putative fusion peptide (amino acids 770–788) into the target cell membrane, and thus forming a stable post-fusion complex [4,5, 6,7]. It has been well documented that binding the S1 subunit to the human Angiotensin-Converting Enzyme 2 (hACE2) receptor acts as a gateway for coronaviral entry inside the host. A stable hot spot is known to consist of a Lys353-Asp38 salt bridge surrounded by four hydrophobic tunnel walls, two each being contributed by the hACE2 receptor and viral S protein, respectively. The stability of viral- receptor interaction is crucial for viral endocytosis and subsequent infection [8]. Any mutation in the viral or receptor domain might curb a stable complex's formation, affecting the infectivity. In this study, we examined 977 S protein sequences deposited across the globe and focused our study on two broad groups, Group-A and B, found primarily in Asia and North America, respectively. These two groups differed at a single amino acid (aa) from aspartate (Group-A) to glycine (Group-B) at position 614, which is close to a highly structurally conserved 194 amino acid long receptor-binding domain (RBD), localized at position 331 - 524 of the S1 subunit [9]. Using homology modeling (HM) and molecular docking, we tried to analyze differences in the viral-receptor binding, which could plausibly affect variants' pathogenicity.

## Materials and Methods:

### Template Search for Modeling of SARS-CoV-2S-protein:

1947 S protein sequences (1273 amino acids long) of SARS-CoV-2 from different geographical regions of the world were retrieved from NCBI (https://www.ncbi.nlm.nih.gov/labs/virus/vssi/#/). Two reference sequences (GenBank accession No. QIU78755 from USA and QIS30405 from Spain) were chosen (one each from two groups [A and B] as per our analysis: in results) for molecular modeling employing the SWISS-MODEL server (https://swissmodel.expasy.org/). Target sequences of S protein belonging to two distinct groups (Group-A and B) of SARS-CoV-2 were searched for selection of most suitable template protein structures by applying standard protein BLAST (Basic Local Alignment Search Tool) database searching algorithm against RCSB protein databank and HHBlits (HMM-HMM–Based Lightning-fast Iterative Sequence Search) searching against SWISS- MODEL template library (SMTL) as of 15-04-2020 with last PDB release on 10-04-2020.

### Model Building, validation, and quality evaluation:

The target-template sequence alignments were performed for finding the highly homologous protein template deposited in PDB for generating the quaternary structure or models for all target sequences. The produced models' geometry was parameterized in a CHARMM27 force field using the OpenMM library of ProMod version 33.0.0 available at the SWISS-MODEL server. The best homology models were selected according to three statistical parameters - Global Model Quality Estimation (GMQE), QMEAN, and quaternary structure quality estimate (QSQE) [10]. The structure validation of modeled proteins was performed through Ramachandran plot (RP) analysis using the SWISS-MODEL structure assessment server to visualize energetically most favored regions for two dihedral angles (Phi (Φ) / (Psi (Ψ)) of backbone amino acid residues.

### Structural comparison of the S protein of two groups of SARS-CoV-2:

The human SARS-CoV-2 S glycoprotein (PDB ID 6VSB) was downloaded from the RCSB protein databank. The pdb format of the modeled SARS-CoV-2 trimeric S proteins (of Group-A and B) were opened in PyMOL Molecular Graphics System, Version 2.0 (Schrodinger, LLC) and superimposed with the native Electron Microscopic (EM) structure of the human SARS-CoV-2 S protein (PDB ID 6VSB) [7]. The structural or amino acid difference in SARS-CoV-2 Group-A S protein with Group- B S protein was observed by superimposing these modeled proteins in PyMOL.

### Molecular docking of human SARS-CoV-2 S protein of two groups with human ACE2 receptor:

We performed three levels of molecular docking of human angiotensin-converting enzyme 2 (hACE2) receptor (PDB ID: 2AJF) [11] with two distinct groups (-A & -B) of human SARS-CoV-2 S protein. Initially, the hACE2 receptor was docked with human trimeric S glycoprotein SARS-CoV-2. Then, SARS-CoV-2 monomeric S protein was docked with the human hACE2 receptor. Finally, the hACE2 receptor was docked with the core RBD (331-524 aa) along with its proximal (from 300-330) and distal (from 525-615) regions of two distinct groups of SARS-CoV-2 S proteins. PatchDock server (http://bioinfo3d.cs.tau.ac.il/PatchDock/), which works on a Geometric Hashing Algorithm (GHA), was used for docking purposes. The default value of 4.0 Å of clustering RMSD for protein-protein docking and default complex type was tuned as the set docking parameters. The PatchDock output lists the top 20 potential docked complexes sorted by geometric shape complementarity (GSC) score and approximate interface (AI) area. The output also shows Atomic Contact Energy (ACE) and 3D transformation, including three rotational angles plus three translational parameters. The protein-protein interaction analysis was performed using the DIMPLOT module of LigPlot+ version 1.4.5. The hydrogen bond formation between docked complexes of hACE2 receptor with RBD of human SARS-CoV-2 S-glycoprotein (Group-A and B) was visualized in the PyMOL Molecular Graphics System, Version 2.0 (Schrodinger, LLC).

## Results and Discussion:

### Modeled S proteins exhibit accuracy and reliability:

**Figure 1:**
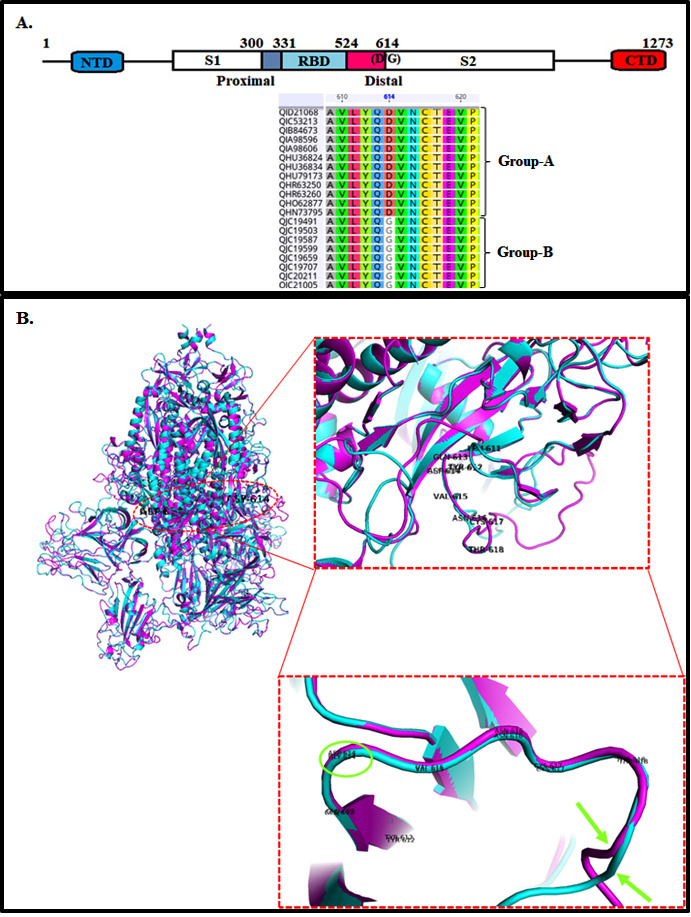
(A) Schematic diagram of S protein's core RBD (331-524 AA) along with proximal (from 300-330) and distal (from 525-615) regions of two distinct groups (-A and -B) of SARS-CoV-2; (B) Ribbon diagram of SARS-CoV-2 Group-A trimeric S protein (Magenta color) superimposed with modeled S protein of Group-B SARS-CoV-2. The enlarged image shows a structural change in variable loop region SARS-CoV-2 S protein o f Group-A concerning the modeled Group-B protein.

A total of 1947 S protein sequences were analyzed from different geographical regions of the world, and 36 prominent mutations were observed. The mutation at 614 amino acid position was the most predominant, with 1105 (56%) had GLY (G), while 842 (44%) had ASP (D) at 614 positions (Figure 1A). A total of 176 protein templates were found in the SMTL based on BLAST, and a total of 676 templates were found by HHblits database searching. Among them, 24 top filtered template structures were found suitable for homology modeling. Ultimately, the human SARS-CoV-2 S glycoprotein (PDB ID 6VSB) was selected as the most suitable template for homology modeling of stars-CoV-2 Group-A and B S proteins. The Group-A and - B S proteins showed 99.26% and 99.17% sequence identity, respectively, and an equal coverage (95%) with the template protein (PDB ID: 6VSB). The high QSQE score and GMQE value of 0.87 and 0.72, respectively, showed the accuracy and reliability of the SARS-CoV-2 Group-A and - B S protein models, deeming them reliable to do structural and docking analysis. Also, a low QMEAN of - 2.81 and -3.10 resonates that the model of SARS-CoV-2 Group-A and - B S protein is of good quality. The number of observed two dihedral angles, i.e., Φ / Ψ pairs, determines the contour lines. In SARS-CoV-2 Group-A and -B S protein 90.49 % and 89.74 %, amino acids except proline or glycine were within the first contour line known as Ramachandran favored region (data not shown). The alignment of the modeled RBDs of human SARS-CoV-2 variants has the same scaffold as the template crystal structure of SARS-CoV-2 (PDB ID: 6vsb). The mutant site D614G is distal to RBD and found in the variable loop region. The core RBD of SARS-CoV-2 S protein is comprised of five major antiparallel β-sheets (β1- β5), three minor β- sheets (β'- β'') and six alpha-helices (α1-α6), which is structurally highly conserved. The modeled SARS-CoV-2 trimeric S protein of Group-A and Group-B was individually superimposed and compared with the native electron microscopic (EM) structure of the SARS-CoV-2 trimeric S protein template (PDB ID: 6VSB). The perfectly superimposed secondary structure elements of modeled proteins with native SARS-CoV-2 trimeric S protein reflect that the modeling is accurate. The ribbon diagrams clearly showed no significant observable structural changes in the modeled trimeric S proteins of group-A with group-B except one amino acid change ASP to GLY at position 614, present in the variable loop region (shown by arrow marks) (Figure 1B).

### S protein variants displayed altered interaction with the receptor hACE2:

**Figure 2:**
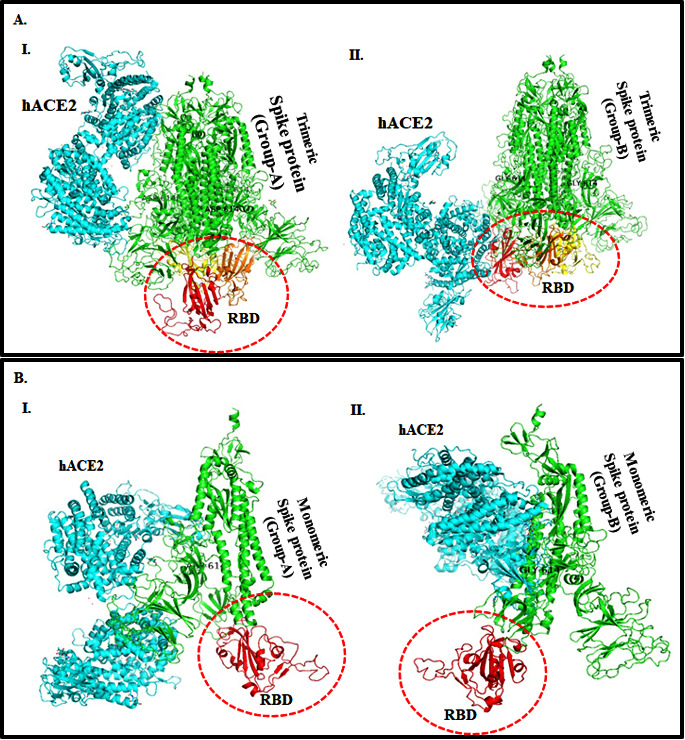
(A) Molecular docking of modeled SARS-CoV-2 S protein with hACE2 receptor (A) Ribbon diagram showing protein-protein interaction between two distinct groups of SARS-CoV-2 trimeric spike protein (green) with hACE2 receptor (cyan) (I) Group-A and (II) Group-B. The RBDs of SARS-CoV-2- S protein from position 331-524 aa is shown in chain A (red), chain B (yellow), and chain C (orange). (B) Ribbon diagram showing protein-protein interaction between two distinct groups of SARS-CoV-2 monomeric spike protein (green) with the hACE-2 receptor (cyan). (I) Group-A and (II) Group-B monomeric SARS-CoV-2 S protein. The RBD of S1 the domain of SARS - CoV-2 spike protein from position 331-524 aa (red).

The molecular docking of hACE2 receptor with SARS-CoV-2 S protein of two distinct groups by PatchDock showed that GSC score (20400) and AI area (3946.10) was maximum for docking complex of hACE2 receptor with RBD of S1 subunit of S protein belonging to Group-B SARS-CoV- 2, while the GSC score (17410) was lowest for docking complex of hACE2 receptor with trimeric S protein of Group-A human SARS-CoV-2. The visualization of the docked complexes of the hACE2 receptor with trimeric Group A S protein showed that hACE2 interacted far away from the RBD (Figure 2A). While the hACE2 interacted in the proximity of the RBDs of Group-B human SARS-CoV-2 S protein (Figure 2A). Similarly, the docked complexes of the hACE2 receptor with monomeric S protein revealed that the hACE2 interacted little away from the RBD of Group-A (Figure 2B) compared to RBDs of Group-B human SARS-CoV-2 S-protein (Figure 2B).

**Figure 3:**
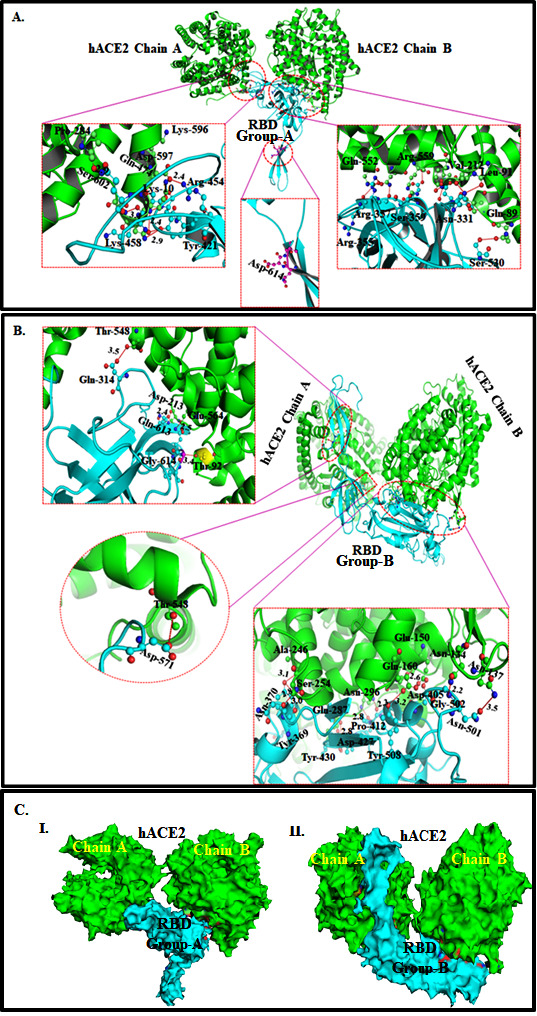
(A) Ribbon diagram of group-A RBD (cyan) of SARS-CoV-2 S protein interacting with hACE2 receptor (green). The interacting amino acid residues are shown in the ball- stick model, and the hydrogen bonds (red broken lines) with bond length are shown in the insets. (B) Ribbon diagram of group-B RBD (cyan) of SARS-CoV-2 S protein i n t e r a c t i n g with hACE2 receptor (green). The interacting amino acid residues are shown in the ball- stick model, and the hydrogen bonds (red broken lines) with bond length are shown in the insets. (C) Surface diagram view of (I) group - ARBD (cyan) and (II) group-B RBD (cyan) of SARS-CoV-2 S protein in the reacting with hACE2 receptor (green). The interfacing amino acid residues are shown in red and blue patches.

The protein-protein interaction analysis revealed that the amino acids of group- A RBD peripherally interacted with two chains (A and B) of the hACE2 receptor. A closer investigation unveils that the RBD of group-A S protein improperly binds inside the cleft of chains A and B of the hACE2 receptor (Figure 3A). Interestingly, it was found that the ASP-614 amino acid containing motif is protruded from the interface (Figure 3C). The interfacing amino acids between group-A RBD and hACE2 that are hydrogen bond-forming (red dots) are shown in Figure 3A.

Interestingly, on the other hand group-B RBD of S protein binds perfectly inside both the domains of chains A and B of the hACE2 receptor with several hydrogen bonds (red dots) and hydrophobic interactions (Figure 3B). The GLY614 amino acid containing motif is embedded in chain A of the hACE2 receptor (Figure 3C) The exact interfacing amino acids between group-B RBD of S protein and hACE2 are shown in Figure 3B.

It was found that 19 aa residues of Group-A RBD interact with 21 aa residues of the hACE2 receptor (Table 1). Out of 19 amino acids of RBD of Group-A S protein, seven amino acids (ARG355, ARG357, THR33, LEU335, SER359, GLU536, and SER530) formed hydrogen bonds with the hACE2 receptor amino acids (GLN552, ASN556, LEU91, GLN338, ARG559, LYS419 and GLN89). Besides, 73 hydrophobic interactions between RBD of Group-A S proteins with 21 aa residues of hACE2 were observed (Table 1). Moreover, this interaction pattern shows that the distal aa residues from core RBD, i.e., LYS529, SER530, GLU536, GLN580, THR581, and LEU582 of Group-A S protein are interacting with the hACE2. It is interesting to note that THR333 and THR500 of RBD strongly interact with the LEU91 and SER370 of hACE2 by forming one hydrogen and several hydrophobic bonds formation in proximity (2.66, 2.97Å) (Table 1).

Analysis of Group B S protein interaction with hACE2 unveils those 34 amino acid residues of core RBD strongly interact (8 hydrogens and 117 hydrophobic bonds) with 35 amino acid residues of the hACE2 receptor (Table 2). Here eight amino acids, i.e., ASN370, TYR508, TYR369, GLU536, THR430, PRO412, ASP427 and THR500 of RBD form eight hydrogen bonds with seven amino acid residues of hACE2 receptor viz., SER254, GLU160, ALA246, LYS419, GLY286, ASN290, and ASN134 (Table 2). Interestingly, only one distal amino acid residue (GLU536) from core RBD was interacting with the LYS419 of hACE2, forming single hydrogen and hydrophobic interaction. More than 100 hydrophobic interactions between amino acids of RBD of Group-B S protein and hACE2 receptor were observed (Table 2). Of interest, the TYR369, THR430, and THR500 residues of RBD strongly interacted with the ALA246, GLY286, and ASN134 of hACE2, the formation of one hydrogen bond (with each) and 30 hydrophobic bonds formation in proximity (Table 2).

The solvent-accessible surface area (SASA) of RBD of Group-A and Group-B SARS-CoV-2 S-protein was 19081.381 and 19307.311 Å2, respectively. The molecular accessible surface area (MASA) of RBD of Group-A and Group-B SARS-CoV-2 was 32482.137 and 32607.174 Å2, respectively (Table 3). The RBD of Group-A SARS-CoV-2 has a relatively smaller SASA in the RBD- hACE2 complex structure than that of Group-B SARS-CoV-2. A slightly larger surface contact area with the hACE2 receptor accompanies the increased SASA and MASA of Group-B SARS-CoV-2. We focused on the region (aa position 611-615) flanking the mutation site D614G. The relative SASA per residue for aa 611-615, i.e., LEU611, TYR612, GLN613, ASP614, and AL615, is 46, 58, 40, 96, and 32% within an overall SASA of 689.913 Å for five residues of group-A RBD. For group B RBD, on the other hand, SASA per residue is 46, 60, 43, 92, and 37%, respectively, within an overall SASA of 547.525 Å (Table 3). It is also noticeable that the solvent-accessible surface area of five distal amino acids of Group- B RBD is lower (547.525 Å) than Group-A RBD (689.913 Å). The ASP614 of Group-A RBD is more accessible (96%) than GLY614 (92%) of group-B RBD of SARS-CoV-2 to the hACE2 receptor. This implies that GLY614 is probably influencing the neighboring aa residues for overall increased accessibility of Group-B RBD to the hACE2 receptor.

## Discussion:

The Spike protein and its RBD have emerged as a mutational hotspot in this novel strain of coronavirus [12]. Recent reports suggest enhanced binding affinity of SARS-CoV-2 to ACE2 receptor compared to SARS-CoV, which is probably responsible for increased infectivity and transmissibility [6,13]. The SARS-CoV-2 S protein adopts a homodimer architecture, of which the RBD undergoes a hinge-like conformational change from perfusion to post-fusion upon binding to the hACE2 receptor. The rotation of trimeric architecture at an angle of 52.2° (determined by aa residues D405-V622- V991) decreases the atomic collision or steric hindrance. It converts ACE2 inaccessible or close or down- conformation of trimeric spike protein into ACE2 accessible or open or up conformation of RBD of trimeric S protein [14]. Studies using computer-aided molecular modeling and protein-protein docking of SARS-CoV-2 spike protein with human ACE2 receptors have shed light on amino acid residues potentially involved in the effective protein-protein interaction [13]. However, the specific actual amino acid residues, which mediate this protein- protein interaction is still unknown. Our in- silico analysis elucidates the interacting amino acid residues between the SARS-CoV-2 RBD of Group- A and B S protein and hACE2 in detail. Through a single aa change at position 614, the S proteins' overall secondary structure showed a similar folding pattern, with no significant structural difference, though minor differences at specific sections of coils or loops were spotted. The structurally preserved functional domains (RBD) and motifs (RBM) in S1 and S2 subunits suggest a highly conserved SARS-CoV-2 S protein structure. However, loop or coil modifications are known to affect the pathogenesis of murine hepatitis virus (MHV) and modulate neurovirulence and invasiveness of human coronavirus (HCoV- OC43) within the central nervous system (CNS) [15,16]. Comparative analysis of structural features and interaction with hACE2 shows that Group-B forms a large binding interface and a larger number of interacting residues than Group-A. Since the trimeric and monomeric forms of spike protein are very bulky in size, it may cause steric hindrance in the visualization of direct interaction of hACE2 receptor to the RBD of S protein. The high-resolution X-ray crystallographic structural data of the SARS-CoV-2 RBD–ACE2 complex unveiled by Lan and their Group show that a total of 17 residues of the RBD are interacting with 20 residues of ACE2 [14]. Our molecular docking shows that 34 amino acid residues of RBD S1 domain of S protein of Group-B SARS-CoV-2 strongly interact with 35 amino acid residues of the hACE2 receptor. Amino acid residues, THR500, ASN501, and GLY502 amino acid residues of RBD of S protein, which interact with the hACE2 receptor, were found common. It is worth noting that their study revealed that THR500 of SARS-CoV-2 RBD forms a 2.6 Å hydrogen bond with Y41 of hACE2, but in our analysis, we found that THR500 forms a 2.18Å hydrogen bond with ASN134 of hACE2. It is interesting to observe that two amino acids THR500 and ASN501, which are under the top five critical residues involved in the interaction with hACE2have been observed in other studies too [14,17,18, 19]. Our docking analysis reveals that the RBD-hACE2 binding interface consists of hydrophobic residues forming an intricate hydrophobic region and hydrogen-bonding network as reported previously [20]. A key mutation at aa position 614 from aspartic acid (D), a polar residue to glycine (G), a nonpolar one in the SARS-CoV-2 S protein, is expected to change the solvent-accessible hydrophobic surface area of the folded protein. This replacement of polar with nonpolar residue resulted in decreased electrostatic energy of Group-B S protein. The amino acid residues LYS529, SER530, GLU536, GLN580, THR581, and LEU582, are distant residues from the RBD (331-524), formed non-specific interaction of RBD of Group- A SARS-CoV-2 to the host hACE2 receptor. On the other hand, Group-B protein is close to the receptor forming a compactly localized interaction compared to Group-A. In a way, it implies a point mutation of ASP614 into GLY614 may have sharply increased the selective affinity of Group-B SARS-CoV-2 RBD to the hACE2 receptor. Single amino acid change enhancing protein interaction efficiency has been observed frequently in other proteins [21]. An insertion of basic aa residues into the loops in the avian influenza virus (H5N1) has reportedly caused its conversion from low pathogenicity to a highly pathogenic state [22]. Also, modifications in S1/S2 cause changes in feline coronavirus (FCoV) pathogenesis [23, 24,25]. It could be hypothesized that the D614G substitution, distal to the receptor-binding motif in the RBD increased the binding capacity of Group B S protein to the human ACE2 receptor, which could be one of the reasons for its increased infectivity and pathogenicity.

## Conclusion:

In conclusion, our study implies that the Group-B S protein having Gly at 614 aa position is structurally more accessible or exposed and binds compactly with hACE2 than the Group-A S protein having Asp at 614. Knowledge of the whole repertoire of residues involved in the interaction between SARS-CoV-2 trimeric S protein (not only core RBD) and hACE2 is needed to properly understand infectivity, transmissibility, and pathogenicity of novel coronaviruses. However, it is essential to note that the S protein mutation may not be the only determining factor for increased transmissibility and infectivity. The combination of mutations in other viral proteins could also affect replication efficiency and other life cycle steps, which need to be examined using the native virus.

## Figures and Tables

**Table 1:** Interacting residues of RBD of Group-A human SARS-CV-2 with amino acids of hACE2 receptor protein

Amino acid residues of RBD of S- protein of Group A SARS-CoV-2	Amino acid residues of Human ACE-2 receptor	Interaction type
ARG355, LYS356	GLN552	1 Hydrogen (2.84 Å) and 7 hydrophobic bonds
ARG357	GLN552, LYS553, ASN556	1 Hydrogen (64.3 Å) and 10 hydrophobic bonds
ASN331	GLY211, VAL212, LEU91	5 hydrophobic bonds
ILE332	LEU91, GLU564, LEU568	3 hydrophobic bonds
THR333	ASN90, LEU91, THR92	1 Hydrogen (2.66 Å) and 21 hydrophobic bonds
ASN334	THR92, SER563, GLU564,	3 hydrophobic bonds
LEU335	THR92, GLN388, ALA387	1 Hydrogen (2.82 Å) and 4 hydrophobic bonds
ASN360	GLU564, ASN572, LEU568, LEU560	4 hydrophobic bonds
CYS361	GLU564	1 hydrophobic bond
SER359	LEU560, ARG559	1 Hydrogen (2.82 Å) and 2 hydrophobic bonds
PRO337	ARG559	6 hydrophobic bonds
GLU340	ALA387, ALA384, ARG559, PHE555	4 hydrophobic bonds
THR581, GLN580, LEU582	ASP213	3 hydrophobic bonds
GLU536	LYS419	1 Hydrogen (2.60 Å) and 2 hydrophobic bonds
LYS529, SER530	GLN89	1 Hydrogen (3.01 Å) and 3 hydrophobic bonds

**Table 2:** Interacting residues of RBD of Group-B human SARS-CoV-2 with amino acids of hACE2 receptor protein.

Amino acid residues of RBD of S-protein of Group B SARS-CoV-2	Amino acid residues of Human ACE-2 receptor	Interaction type
ALA372	TYR255, SER254, PRO253	6 hydrophobic bonds
SER371	SER254, PRO253	2 hydrophobic bonds
ASN370	SER254, PRO253	1 Hydrogen (3.07 Å) and 2 hydrophobic bonds
VAL503	GLU160	1 hydrophobic bond
TYR508	GLU160, ASP157	1 Hydrogen (3.02 Å) and 3 hydrophobic bonds
GLY504	GLU160, ILE151	2 hydrophobic bonds
ASP405	ILE151, GLU150, ASN154	3 hydrophobic bonds
ARG408	GLU150, ALA153, ASN154	3 hydrophobic bonds
PHE374	PRO253, TYR252, ALA251	3 hydrophobic bonds
ASN437	ASP157	1 hydrophobic bond
TYR369	ALA246, ASN250	1 Hydrogen (2.83 Å) and 20 hydrophobic bonds
PRO384	ASN250, LYS247	2 hydrophobic bonds
SER375	TYR252, ASP157, ALA251, TYR158	4 hydrophobic bonds
SER383	LYS247	1 hydrophobic bond
VAL407	LEU156	1 hydrophobic bond
PHE377	ASN250, ALA251, LEU281	3 hydrophobic bonds
THR376	ALA251, LEU256, LEU281	3 hydrophobic bonds
LYS387	LEU156, SER280, LEU281	3 hydrophobic bonds
GLY381	PRO284, PHE285, GLN287, GLY286	4 hydrophobic bonds
GLU536	LYS419	1 Hydrogen (2.60 Å) and 1 hydrophobic bond
THR380	SER280, PHE285, LEU281, GLY286,	4 hydrophobic bonds
GLY431	GLN287	1 hydrophobic bond
ASP428	GLN287, GLY286	2 hydrophobic bonds
THR430	GLN287, GLY286	1 Hydrogen (2.92 Å) and 1 hydrophobic bond
GLN414	ASP295	1 hydrophobic bond
PHE429	GLN287, GLY286, LYS288	3 hydrophobic bonds
PRO412	ASP295, ASN290	1 Hydrogen (2.29 Å) and 1 hydrophobic bond
THR415	VAL298, ASP295, THR294	3 hydrophobic bonds
ASP427	PRO289, ASN290	1 Hydrogen (30.2 Å) and 1 hydrophobic bond
CYS379	VAL283	1 hydrophobic bond
ASN501	ASN137, GLU140, ASP292	3 hydrophobic bonds
GLY502	GLU140, ASN134	2 hydrophobic bonds
GLY413	ASP295, THR294, ASN290, ASP292	4 hydrophobic bonds
THR500	ASN14, ASP136	1 Hydrogen (2.18 Å) and 8 hydrophobic bonds

**Table 3:** Percentage Solvent accessible surface area (SASA) of distal amino acids from core RBD and its effect on an overall molecular and solvent accessible surface area of RBD.

SARS-CoV-2 S Protein	Solvent accessible surface area (SASA) of RBD (Å2)	Molecular accessible surface area (MASA) Of RBD (Å2)	Name of distal amino acids from core RBD	Percentage Solvent accessible surface area (%)	Overall Solvent accessible surface area (SASA) of five distal amino acid residues (Å2)
Group-A	19081.381	32482.137	LEU611	46	689.913
			TYR612	58	
			GLN613	40	
			ASP614	96	
			VAL615	32	
Group-B	19307.311	32607.174	LEU611	46	547.525
			TYR612	60	
			GLN613	43	
			GLY614	92	
			VAL615	37	

## References

[1] Ashour HM (2020). Pathogens.

[2] Zhu N (2020). The New England Journal of Medicine.

[3] Dong E (2020). The Lancet. Infectious Diseases.

[4] Andersen KG (2020). Nature Medicine.

[5] Du L (2009). Nature Reviews. Microbiology.

[6] Walls AC (2020). Cell.

[7] Wrapp D (2020). Science.

[8] Wu K (2011). Journal of Virology.

[9] Zhang Y (2005). Computational biology and Chemistry.

[10] Studer G (2020). Bioinformatics.

[11] Li F (2005). Science.

[12] Sheikh JA (2020). Infection genetics and evolution.

[13] Wan Y (2020). Journal of Virology.

[14] Lan J (2020). Nature.

[15] Frana MF (1985). Journal of Virology.

[16] Le CA (2015). PLoS pathogens.

[17] Wong SK (2004). The Journal of Biological Chemistry.

[18] Yan R (2020). Science.

[19] Zhou P (2020). Nature.

[20] Wang Y (2020). Proceedings of the National Academy of Sciences of the United States of America.

[21] Selvandiyan A (1995). FEBS letters.

[22] Chen J (1998). Cell.

[23] Andre NM (2019). JFMS open reports.

[24] Jaimes JA (2020). Viruses.

[25] Licitra BN (2013). Emerging Infectious Diseases.

